# Experiences in outpatient parenteral antimicrobial therapy (OPAT): Barriers and challenges from the front lines

**DOI:** 10.1017/ash.2021.213

**Published:** 2021-11-03

**Authors:** Nicole Ng, Pamela Bailey, Rachel Pryor, Lillian Fung, Christine Veals, Kenneth Sabouri, Julie Reznicek

**Affiliations:** 1 Virginia Commonwealth University, School of Medicine, Richmond, Virginia; 2 Division of Infectious Diseases, Department of Internal Medicine, Virginia Commonwealth University Health System, Richmond, Virginia; 3 Hospital Epidemiology and Infection Control, Virginia Commonwealth University Health System, Richmond, Virginia; 4 Option Care Health, Ashland, Virginia

## Abstract

**Background:**

Outpatient parenteral antimicrobial therapy (OPAT) is now the standard of care for managing patients who no longer need inpatient care but require prolonged intravenous antimicrobial therapy. OPAT increases patient satisfaction, reduces the lengths of hospital stay, lowers emergency department readmission rates, and decreases total healthcare spending.

**Objective:**

To investigate Virginia Commonwealth University Health System’s experience with OPAT and to highlight the obstacles patients and clinicians face when navigating and utilizing this program.

**Design:**

We conducted this descriptive study at a large, academic, tertiary-care hospital in Central Virginia.

**Methods:**

We performed manual reviews of electronic medical records of 602 patient, and we evaluated the records of those receiving OPAT between 2017 and 2020. Reviews included antimicrobial agents, diagnoses requiring OPAT, adverse effects related to antimicrobials, adverse effects related to peripherally inserted central catheters (PICC), readmission rate, discharge destination, and death. We evaluated our program with descriptive statistics.

**Results:**

Among 602 patients who received OPAT, most were diagnosed with bacteremia or musculoskeletal infections. Patients were either discharged home or to another healthcare facility, with the former comprising most of the rehospitalizations. Ertapenem and vancomycin were associated with the most adverse drug events among our cohort. Elevated transaminase levels were noted in 23% of patients. The rate of PICC-line adverse events in this study population was 0.05%.

**Conclusions:**

Our findings highlight the barriers and challenges that patients and providers face when receiving OPAT, and they can inform efforts to improve patient clinical outcomes.

Outpatient parenteral antimicrobial therapy (OPAT) is now the standard of care for managing patients who no longer need acute care but require prolonged intravenous antimicrobial therapy. OPAT can be administered at home, in an infusion center, or in a skilled nursing facility (SNF); >85% of patients utilizing the home-based model.^1^ A newer term, “COpAT” or “complex outpatient antimicrobial therapy,” is evolving to describe the increasingly high-stakes nature of treating some infections in the outpatient setting.^2^

OPAT has repeatedly proven to reduce lengths of hospital stay, decrease total healthcare spending, lower emergency department readmission rates, and increase patient satisfaction.^2–4^ Prolonged hospitalization carries a ∼5.5% risk of adverse drug reaction and a 17.6% risk in infection, according to a model created and published by Hauck and Zhao.^5^ When patients eligible for OPAT finish therapy elsewhere, more hospital beds are available, which also shortens emergency department waiting times and increases the capacity to accept patient transfers.^6^ In Belgium, 152 OPAT treatments resulted in avoidance of 3,153 days of hospitalization.^7^ The critical importance of hospital capacity, especially flexibility and adaptability, is particularly highlighted in the era of the SARS-CoV-2 pandemic.^6^ Because inpatient care accounts for one-third of all healthcare spending in the United States,^6^ utilizing subacute healthcare programs such as OPAT can bring many financial benefits to healthcare systems.

Although variability exists among these settings, OPAT can be administered in 3 main locations: home, an infusion center, or an SNF. The Infectious Diseases Society of America (IDSA) has published guidelines, and other research groups have offered their recommendations for a successful OPAT team.^8^ The 3 main team members are an infectious diseases (ID) specialist or consultant to spearhead the team’s efforts, a clinical nurse who oversees patient education and ensures monitoring of laboratory test results, and a clinical pharmacist who is responsible for monitoring drug safety.^9^ Each role is critical in preventing adverse events. During a typical OPAT course, adverse events range from 6% to 44%, including adverse drug events (ADEs) and vascular-access complications.^4,7^ In a large UK study of 2,870 patient episodes, rates of drug and central-line events were 3.3 and 1.78 per 1,000 treatment days, respectively. Greater odds of OPAT success were observed among patients on longer therapy (>14 days; OR, 2.32; *P* < .01), patients utilizing a peripheral line (OR, 1.83; *P* < .01), patients who were treated in the clinic compared with self-administration (OR, 2.1; *P* < .02), and patients who did not experience an adverse event (OR, 0.23; *P* < .01).^10^ Readmission rates for OPAT patients vary and can be as high as 20%–30%, but established OPAT programs note rates of 10%–20%.^2^

Despite the clear strengths of this therapy modality, OPAT also harbors challenges. Due to the reduction of immediate clinical supervision with OPAT, close monitoring of patients is needed to ensure positive patient outcomes. Programs can struggle with readmission rates and other safety metrics,^11,12^ as responsibility shifts from hospitals to patients receiving OPAT. In a survey by the Emerging Infection Network, 672 members responded with a theme of lack of support: data analysis, information technology, financial assistance, and administrative assistance specifically. Overall, these responses were summarized by a lack of institutional support for this modality of therapy.^13^ However, research regarding the challenges related to this new modality of therapy is still minimal.

VCUHS is an academic tertiary-care facility in an urban setting, with 865 inpatient beds. At VCUHS, we formalized our OPAT program in 2017. Approximately 60 patients per month are discharged to receive OPAT. In 2019, we transitioned away from a paper record-keeping system to an electronic database for OPAT management. These electronic records are kept in RedCap to ensure smoother follow-up and to ensure that patient name, location, antimicrobial regimen, and laboratory results are easy to access.

Our OPAT program supports 11 ID physicians and consists of a medical director (ID physician), 2 nurses, and a pharmacist. The ID physician who discharges the patient is responsible for the laboratory results and follow-up appointments; however, the medical director is available for the immediate review of results if the ordering physician is not available. The medical director also develops and improves protocols, disseminates information to the SNFs, and regularly meets with the team to identify potential areas for improvement.

## Methods

Since 2017, we have discharged >2,000 patients from VCUHS with OPAT. Using records stemming from OPAT monitoring, we created a database in RedCap (Vanderbilt, TN) and manually reviewed all 602 records from January 2017 to March 2020 available within our electronic medical record. There were OPAT records without clear medical record numbers and thus these patients were excluded from study. We looked at antimicrobial agents, diagnoses requiring OPAT, adverse effects related to antimicrobials, adverse effects related to peripherally inserted central catheters (PICC), all-cause readmission rate, discharge destination, and death. Readmissions were considered if patient was readmitted while on OPAT. Death was characterized as related to the infection or not. Diagnoses were categorized for analysis (Table 1). Patients in this study only were evaluated using their primary diagnosis for OPAT enrollment. We evaluated our program with descriptive statistics. Diagnoses and antimicrobial regimens were extracted from the ID consultants’ notes.

**Table 1.** Categorization of Infections Treated with outpatient parenteral antimicrobial therapy (OPAT)

Category Title	Diagnoses Included Within Category
Osteomyelitis	Osteomyelitis, appendicular, long bone, rib, thumb, calcaneus, foot, toes, sternoclavicular, vertebral, skull, pelvic, mandibular
	Discitis
Bacteremia	Neutropenic fevers
	Central-line–associated bloodstream infection
Skin and soft-tissue infection	Cellulitis/Skin abscess
	Pressure injuries
	Mastitis/Breast abscess
	Surgical site infections
	Infected hematoma
	Tenosynovitis
	Septic thrombophlebitis
	Myositis
Prosthetic joint infection (PJI)	
Septic arthritis	
Pleural pulmonary infection	Pneumonia
	Empyema
	Pleural effusion
	Invasive aspergillosis
	Pulmonary sarcoid with positive culture
	Pulmonary septic emboli
Endocarditis/Endovascular infection	Vascular graft infections
Complicated urinary tract infection (UTI)	Pyelonephritis
	Prostate abscess
	Catheter-associated UTI
Central nervous system (CNS) infections	Brain abscess
	Meningitis
	Neurosyphilis
Cardiac device infection	Left ventricular assist device (LVAD) infection
	Pacemaker infections
Orofacial space infections	Masticator space abscess
	Dental abscess
	Submental abscess
	Ludwig’s angina
Device infection	Video-assisted thoracoscopic surgery (VATS) infection
	Ventriculoperitoneal (VP) shunt infection
	Deep brain stimulator (DBS) infection
	Baclofen pump infection
	Spinal cord stimulator infection
Fungemia	
Cytomegalovirus (CMV) infection	
Other	Disseminated histoplasmosis
	Mediastinal mass
Pericarditis/Myocarditis/Mediastinitis	

## Results

**Fig. 1.**
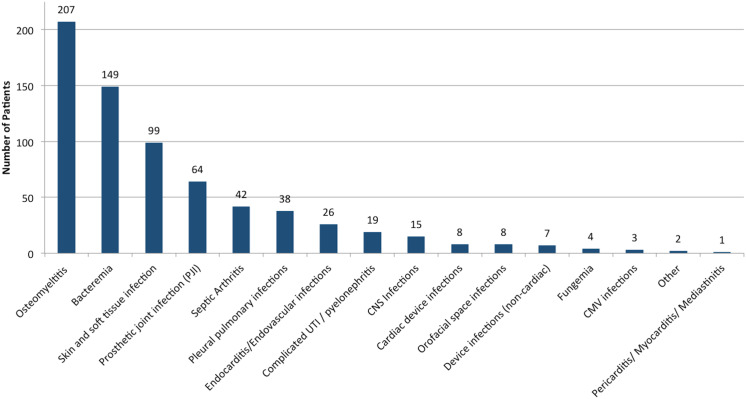
Common diagnoses in patients utilizing OPAT. Common diagnoses in 602 patients examined. “Other” diagnoses include mediastinal mass and disseminated histoplasmosis. Note. CMV, cytomegalovirus; UTI, urinary tract infection.

Most patients (n = 207, 34.4%) had osteomyelitis, including long bones, foot, vertebrae, and other sites. Other common diagnoses included bacteremia (n = 149, 24.8%), skin and soft-tissue infections (SSTIs, n = 99, 16.4%), prosthetic joint infections (PJIs; n = 64, 10.6%), septic arthritis (n = 42, 7.0%), pleural pulmonary infections (n = 38, 6.3%), and other infections (n = 93, 15.4%) including endocarditis or endovascular infections, urinary tract infections (UTIs) or pyelonephritis, and central nervous system infections (Fig. 1).

Moreover, 469 OPAT patients (78.0%) were discharged home with home health services. Others were discharged to an SNF (n = 68, 11.3%), long-term acute-care hospital (n = 36, 6.0%), or daily infusion center (n = 25, 4.2%). During their OPAT courses, 84 patients were rehospitalized; 31 (27.9%) of these had bacteremia or sepsis and 19 (17.1%) had an SSTI. The percentages of other patients who were rehospitalized were proportional to the frequencies of the diagnoses. Among all patients who were rehospitalized, 85.7% were initially discharged home.

**Fig. 2.**
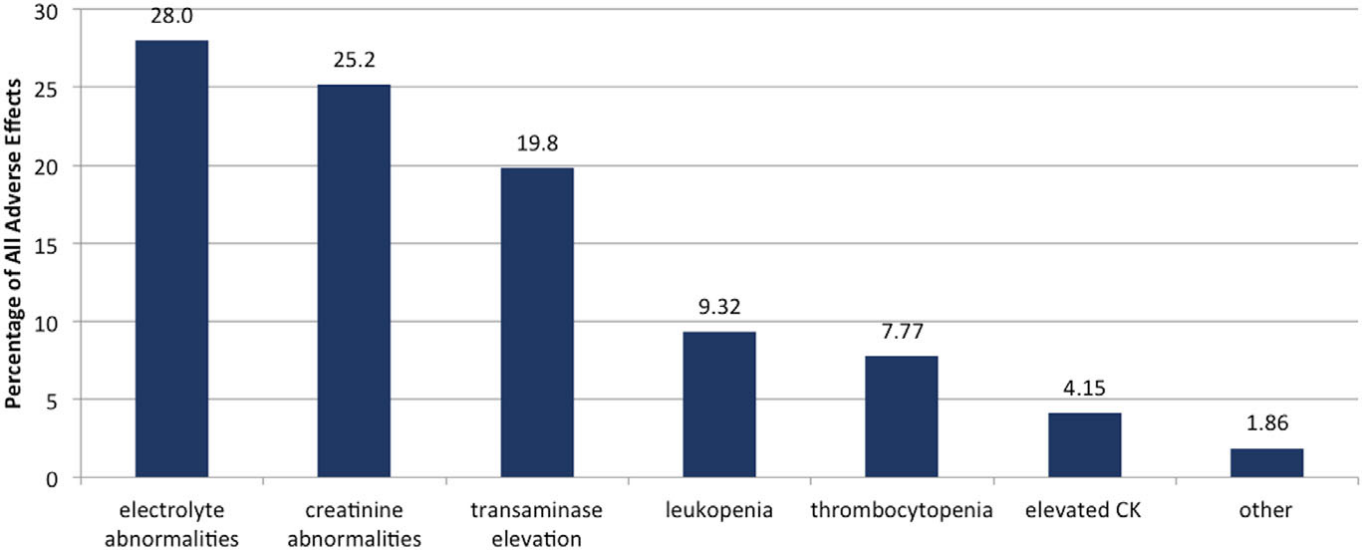
Distribution of adverse drug events. Other includes rash, nausea, diarrhea, hearing loss. There were no cases of vomiting, anaphylaxis, or vancomycin hypersensitivity. Note. CK, creatinine kinase.

The most commonly prescribed antibiotics were ertapenem (16.1%), vancomycin (16.0%), ceftriaxone (12.7%), daptomycin (11.0%), and piperacillin/tazobactam (9.9%). Among our 602 patients, 69.1% were receiving 1 antibiotic, 25.7% were receiving 2 agents, and 5.2% were receiving ≥3 agents. Electrolyte abnormalities occurred in 54.2% of patients. Other significant adverse effects included creatinine abnormalities (48.3%), transaminase elevation (35.2%), and leukopenia (17.6%). Of all ADEs, electrolyte abnormalities and creatinine abnormalities were the most frequent (Fig. 2).

**Fig. 3.**
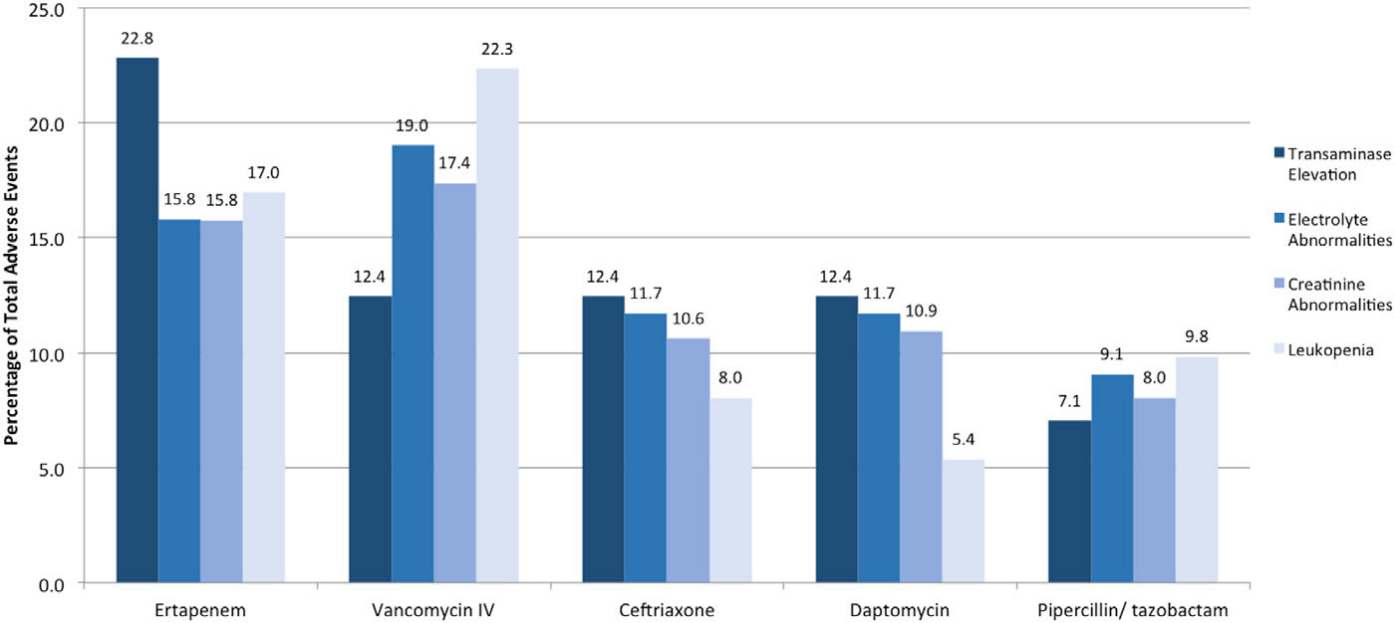
Various antibiotics and their proportion of adverse drug events.

Patients on ertapenem or vancomycin experienced the most ADEs overall (Fig. 3). Patients on ertapenem had the most cases of elevated transaminases, whereas patients on vancomycin exhibited the most leukopenia, creatinine changes, and electrolyte abnormalities. Of the patients on ertapenem, 63.3% were only receiving 1 antimicrobial. Of 83 people on daptomycin, 40 patients had abnormal creatine kinase levels. In this study, 35 patients (5.8%) did not have documented laboratory results.

In total, 30 PICC-related events were identified in this study. Dislodgement was the most common PICC-related adverse event (n = 23, 76%). Others included local PICC-line site reaction (n = 4, 13.3%), central-line–associated bloodstream infection (n = 2, 6.7%), and deep vein thrombosis or thrombophlebitis (n = 1, 3.3%). Notably, 93% of adverse PICC-line events occurred in patients who were discharged home.

Most patients completed their OPAT course on time (n = 454, 75.4%). Nevertheless, 14.2% of patients prematurely terminated their therapy program either due to rehospitalization, intolerance to medication, or nonadherence. Upon termination due to adverse effects, 17 patients (2.8%) required an oral antibiotic ‘tail.’ Other patients either required a longer intravenous course (n = 19, 3.2%), were lost to follow-up (n =1 8, 3.0%), or passed away from infectious or other causes (n = 9, 1.5%). Although most patients who were discharged home were able to complete their antimicrobial therapy on time (n = 358, 76.3%), 15.0% of these patients who went home terminated their OPAT course prematurely and 4.0% required a longer course. Amongst those who were discharged to an SNF, 17.6% had ended therapy prematurely. Among those who were discharged to a long-term acute-care hospital, 5.6% of patients terminated therapy.

## Discussion

Outpatient antimicrobial parenteral therapy is an increasingly popular therapy modality for patients who need intravenous antimicrobial therapy without acute care, but it is not a benign intervention despite the known benefits. Specific challenges include increased need for monitoring, personal responsibility of the patients to administer antibiotics, and need for institutional support.^1^

In our retrospective study, the most common diagnoses requiring long-term antimicrobials were bacteremia and musculoskeletal infections, and most patients were discharged home with home health services. Although we cannot make inferences on causation, the trend that our patients discharged home were readmitted more frequently than those discharged elsewhere may indicate that patients need more or better home health support. This support could also be provided by telehealth, with an ability to check in on patients more frequently to allow for earlier inteventions before major problems occur.^1^

We noted significant ADEs related to ertapenem and vancomycin, which were 2 of the most-prescribed antibiotics and the agents with the most overall ADEs (Fig. 3). Previous studies have highlighted a favorable safety profile for ertapenem, so additional studies will be needed to further evaluate the ADEs in our cohort of patients.^15,16^ Ertapenem is widely used due to its favorable once-daily administration convenient for OPAT; however, this convenience may need to be reassessed in light of ADEs.

Compared to other intravenous antimicrobials, vancomycin is notoriously more difficult to manage in the outpatient setting because of the need to closely monitor drug levels, which affect dosage and frequency of administration. This factor relates to a higher rate of ADEs both in our study and in other studies, often leading to a change in the antimicrobial regimen or to early discontinuation of therapy.^4^ Also, therapeutic implications are associated with obtaining vancomycin trough levels. If the trough is obtained at the incorrect time or there is a delay in sending out the adjusted vancomycin dose, patients may experience days of subtherapeutic treatment. We were not able to find any evidence to support difficulties with obtaining vancomycin levels in the outpatient setting. Among internal data, of 218 vancomycin levels drawn, only 34% fell within the target therapeutic range of 15–20 mcg/L (unpublished data). This finding further supports the need for the use of other anti-MRSA agents that do not require serum-level monitoring (ie, daptomycin), though other factors, such as antimicrobial stewardship and cost or insurance coverage, may need to be considered.

The IDSA guidelines provide recommendations for laboratory monitoring for specific antimicrobials that may be used in OPAT.^8^ Our study spanned 2017–2019, with publication of the guidelines in 2018. Thus, for a large portion of our patients, monitoring was conducted at the physician’s discretion. Transaminase elevation was the third most common ADE, occurring in 35% of patients. Transaminase elevation may have been related to the underlying infection or secondary to other medications (ie, acetaminophen), but it is worth considering when developing a laboratory monitoring plan for a patient. However, this factor may be a moot point in future studies because the guidelines are adapted into clinical practice. A balance exists between the guideline recommendations, diagnostic stewardship, and unnecessary laboratory tests, as well as the individual patient. Regardless, Keller et al^17^ reported that following the introduction of an ID-based transition of care for OPAT patients, compared to patients without ID-based care, receipt of laboratory test results was higher (increasing from 37.4% to 94.3%). This finding supports the importance of good teamwork in OPAT.

The rate of adverse effects related to PICC lines in our population was 0.05%. This result is significantly lower than those reported in other studies, though many other studies use days present as evaluation for PICC-related side effects. This low rate may reflect uncaptured events in patients who were rehospitalized. It may reflect robust hospital epidemiology interventions targeted at preventing central-line–associated bloodstream infection, in addition to patient education prior to discharge. In a study of emergency department visits related to OPAT, the most common reason for emergency department visit was vascular-access complications (n = 104), with 53 visits related to occlusion and 19 related to dislodgement.^18^ Few PICC events related to those who went to daily infusion centers or other healthcare centers, and this could be a consideration for some patients who may struggle with PICC care at home though this comes with its own challenges with transportation to infusion centers.

In our OPAT experience, 84 patients were rehospitalized; most of whom were initially discharged home (85.7%). Although this is a reasonable complication rate and we cannot infer causation in this retrospective study, it could be improved. Others note that outpatient follow-up within 2 weeks was associated with lower risk of all-cause 30-day readmission (adjust odds ratio = 0.33; *P* = .0001),^19^ but short-term follow-up is not always feasible for the both the patient and the physician. The role of telehealth visits to “check in” on OPAT patients on a weekly or biweekly bases needs to be explored further. Tan et al^14^ have shown telehealth to be a safe and efficacious way to monitor geographically remote patients in their study of 88 OPAT encounters. We did not evaluate ID follow-up appointments nor the correlation with 30-day readmissions. Studies have demonstrated that improved management of OPAT (ie, led by an ID physician) can reduce the probability of readmission or emergency department visits. Shah et al^20^ found that in OPAT programs led by ID physicians, patients not only had lower 30-day hospital admission rates but also paid less in total healthcare payments.^20^ OPAT programs led by ID physicians have also been reported to have more frequent laboratory testing and follow-up as well as fewer errors in prescribing.^17^

Our study had several limitations. We lacked data from hemodialysis centers because we do not follow patients there after providing initial recommendations. Not all OPAT records contained patient medical record numbers; therefore, some patients were excluded. This study was conducted at a single center, which may limit generalizability. We also lacked data in 3% of patients who were lost to follow-up, and these patients may have had significant adverse effects, such as rehospitalization, ADEs, or death, which are not captured and thus may have skewed the data. Furthermore, the IDSA guidelines for OPAT came out in the midst of our study period, which affected clinical practices regarding OPAT in ways that we cannot account for in this retrospective review.

In conclusion, we examined >600 patients utilizing OPAT in this study, and we observed that most of our rehospitalized patients were initially discharged home. Ertapenem and vancomycin were associated with the most ADEs among our cohort, which was unexpected for ertapenem due to its simple dosing schedule and favorable safety profile. We noted transaminase elevation in 23% of patients, which may suggest that measuring liver function needs to be considered for medication monitoring. Our rate of PICC-line adverse events in our study population was significantly lower than those in other studies, and most occurred in those who were discharged home.

In this dynamic healthcare landscape, we must continue to explore ways to optimize surveillance of our OPAT patients. We must always strive to minimize adverse effects and other barriers that prevent us from achieving our therapeutic goals.

## Research transparency and reproducibility

Access to our database will be provided upon request.
